# Gastrointestinal Mucosal Disruptions During ART-Treated SIV*/Plasmodium fragile* Co-Infection

**DOI:** 10.20411/pai.v11i1.854

**Published:** 2026-02-03

**Authors:** Sydney M. Nemphos, Hannah C. Green, James E. Prusak, Sallie L. Fell, Cecily Midkiff, Avelina Rodgers, Jillian Perret, Kelly Goff, Jordyn Miller, Megan Varnado, Kaitlin Didier, Natalie Valencia, Matilda J. Moström, Coty Tatum, Mary B. Barnes, Clara E. Krzykwa, Lori A. Rowe, Carolina Allers, Brooke Grasperge, Kristina De Paris, Nicholas J. Maness, Amitinder Kaur, Berlin Londono-Renteria, Robert V. Blair, Jennifer A. Manuzak

**Affiliations:** 1 Division of Immunology, Tulane National Biomedical Research Center, Covington, Louisiana; 2 Division of Microbiology and Immunology, Tulane University School of Medicine, New Orleans, Louisiana; 3 Division of Comparative Pathology, Tulane National Biomedical Research Center, Covington, Louisiana; 4 Division of Microbiology, Tulane National Biomedical Research Center, Covington, Louisiana; 5 Division of Veterinary Medicine, Tulane National Biomedical Research Center, Covington, Louisiana; 6 Department of Microbiology and Immunology, University of North Carolina School of Medicine, Chapel Hill, North Carolina; 7 Department of Tropical Medicine and Infectious Disease, Tulane University Celia Scott Weatherhead School of Public Health and Tropical Medicine, New Orleans, Louisiana

**Keywords:** Disease Models (Animal), Infectious Diseases, HIV, HIV Co-infection, Simian Immunodeficiency Virus, Malaria, Plasmodium, Gastrointestinal Tract, Innate Immunity, Neutrophils, Neutrophil Extracellular Traps

## Abstract

**Background:**

Human immunodeficiency virus (HIV) and *Plasmodium* spp., which causes malaria, are co-endemic. Previously, we showed that during antiretroviral therapy (ART)-treated simian immunodeficiency virus (SIV)/*Plasmodium fragile* co-infection, peripheral markers of neutrophil extracellular trap (NET) formation positively correlated with peripheral markers of disease and gastrointestinal (GI) dysfunction. However, the impact of co-infection directly in the GI mucosa is unclear. We hypothesized that ART-treated SIV/*P. fragile* co-infection would result in peripheral and GI immune disruption associated with exacerbated clinical manifestations of SIV and *P. fragile*.

**Methods:**

Adult male rhesus macaques (RMs; n=6) were inoculated with SIVmac239, initiated ART at week 8 post-SIV infection (p.i.), were inoculated with *P. fragile* at week 12 p.i., and were followed until week 20 p.i. Plasma viral loads, peripheral parasitemia, and peripheral and GI immune cell frequencies and function were assessed longitudinally.

**Results:**

We observed significant CCR5+ CD4+ T cell decline in the periphery, colon, and duodenum following SIV infection. Neutrophil frequencies were unchanged throughout ART-treated SIV/*P. fragile* co-infection. Notably, duodenum NET-forming granulocyte frequencies were significantly positively associated with peripheral SIV burden following *P. fragile* co-infection but were unassociated with peripheral parasitemia and CD4+ T cell frequencies. Finally, although *P. fragile* was present in the duodenum, GI parasite burden was not associated with NET-forming granulocyte frequencies, peripheral viral loads, or CD4+ T cell frequencies.

**Conclusions:**

*P. fragile* co-infection during ART-treated SIV could cause mucosal disruptions that contribute to peripheral SIV replication despite ART. These data may have implications for HIV and malaria disease progression and treatment strategies.

## INTRODUCTION

Among the 39.9 million people living with HIV (PWH) in 2023, there were 1.3 million new cases and over 630,000 HIV-related deaths [1]. Antiretroviral therapy (ART) enables sustained viral load suppression, CD4+ T cell recovery, and improved quality of life [2]. However, even with perfect adherence, ART does not eliminate the viral reservoir; plasma viremia rapidly rebounds, and CD4+ T cell counts decrease following ART interruption or cessation [3]. Similarly, malaria, caused by infection with *Plasmodium* spp., is a global health burden, causing over 263 million cases and over 597,000 malaria-related deaths in 2023 [4]. Antimalarial drugs are effective and serve as preventative and curative treatments for *Plasmodium* spp. infection, but the development of drug resistance undermines their lasting effectiveness [5].

HIV and malaria are co-endemic in similar geographical locations, with a co-incidence of 43% [6]. Previous work has identified a reciprocally antagonistic relationship between HIV and *Plasmodium* that results in increased transmission and pathogenesis of both pathogens [7–10]. For example, *Plasmodium*-infected PWH exhibited higher HIV viral loads compared to malaria-naïve individuals, suggesting that malaria co-infection in PWH exacerbates viral replication, potentially increasing the likelihood of HIV transmission risk [7, 8, 11, 12]. Likewise, HIV infection is associated with increased malaria severity and malaria-induced mortality [9, 13].

A possible mechanism underlying enhanced disease pathogenesis during HIV/malaria co-infection is uncontrolled inflammation [14, 15]. PWH exhibit increased inflammation and immune activation that persist despite ART and are associated with viral replication and increased risk of acquiring co-infections [16, 17]. Similarly, *Plasmodium* spp. infection induces a pro-inflammatory response linked with increased risk of malaria-associated morbidity and mortality [15]. Importantly, these observations on HIV/*Plasmodium* spp. co-infection have been made in ART-naïve PWH, and the impact of ART on HIV/*Plasmodium* spp. interactions, especially in critical tissue sites, remains incompletely defined. An enhanced understanding of the underlying mechanisms of HIV and malaria disease pathogenesis in the context of co-infection and suppressive ART will support the development of more efficacious treatments and therapeutics that reduce the risk for HIV- and malaria-associated morbidity and mortality [18, 19].

The gastrointestinal (GI) mucosa is critical in maintaining GI homeostasis, both as a physical barrier and as an immune-rich landscape. Indeed, the GI tract contains approximately 5×10^10^ immune cells, made up primarily of lymphocytes, including T cells, B cells, and plasma cells, as well as other immune subsets, like mast cells and granulocytes [20]. Untreated HIV infection results in massive depletion of mucosal CD4+ T cells, leading to greater GI barrier permeability [21–24] and microbial translocation, which further skews the GI immune landscape towards a pro-inflammatory environment [25, 26]. GI dysfunction in PWH is not completely ameliorated by ART [27, 28].

The GI tract is also a site of *Plasmodium* spp. sequestration, which is associated with loss of barrier integrity, elevated microbial translocation, and increased risk of severe manifestations of malaria [29–31]. Our previous work suggests that co-infection of rhesus macaques (*Macaca mulatta*; RMs) with simian immunodeficiency virus (SIV) and *P. fragile*, which model HIV and *P. falciparum*, respectively [32–36], results in increased levels of peripheral markers of GI barrier permeability and microbial translocation [37]. Notably, these peripheral markers of GI dysfunction were significantly and positively correlated with elevated plasma markers of neutrophil extracellular trap (NET) formation [37]. However, the effects of HIV/*Plasmodium* spp. co-infection directly in the GI mucosa have not been well defined. Here, we hypothesized that *P. fragile* co-infection in ART-treated SIV-infected RMs would result in peripheral and GI immune disruptions that would be associated with exacerbated clinical manifestations of both SIV and *P. fragile* infection. To test this hypothesis, we longitudinally monitored clinical, immunological, and disease parameters in the periphery and GI mucosa throughout *P. fragile* co-infection of ART-treated SIV-infected RMs.

## METHODS

### Study Animals and Approval

Six adult (aged 4-12 years) male Indian-origin RMs were housed and cared for at the Tulane National Biomedical Research Center (TNBRC; Office of Laboratory Animal Welfare Assurance Number A4499-01). Procedures were performed in Association for Assessment and Accreditation of Laboratory Animal Care accredited facilities (AAALAC Number 000594) compliant with the United States Department of Agriculture regulations, the Animal Welfare Act (9 CFR), the Animal Care Policy Manual, the National Research Council in the Guide for the Care and Use of Laboratory Animals, and the Weatherall Report. All RMs had no previous exposure to either SIV or any *Plasmodium* spp. and were negative for MHC class I alleles associated with SIV control (Mamu-A*01, Mamu-B*08, and Mamu-B*17) [38–40]. Four RMs (RM21-0030, RM21-0031, RM21-0032, RM21-0033) were singly housed, and RM23-0178 and RM23-0179 were co-housed indoors with a 12-hour/12-hour light/dark cycle under climate-controlled conditions.

Animal welfare was monitored daily, and all abnormalities were reported to a veterinarian. RMs had access to water ad libitum, and their diet consisted of commercial monkey chow (Purina LabDiet; PMI Nutrition International), supplemented with fruits, vegetables, and foraging treats. Four RMs received antibiotics, and 1 received a blood transfusion, as previously described [37]. All procedures, including anesthesia, which consisted of ketamine hydrochloride (Ketaset 6 mg/kg; Zoetis) and buprenorphine (0.01 mg/kg), injected intramuscularly, were performed under TNBRC veterinarian direction in accordance with TNBRC policy and the Weatherall Report. Euthanasia was conducted using methods recommended by the American Veterinary Medical Association and per IACUC recommendations.

### SIV Inoculation, Monitoring, and ART Treatment

RMs were inoculated intravenously (i.v.) with 50 TCID50 SIVmac239 [41]. Plasma viral loads were monitored weekly via RT-qPCR (lower limit of detection=83 copies/mL) [42]. At week 8 post-SIV infection (p.i.), RMs initiated subcutaneous (s.c.) daily ART treatment, consisting of tenofovir disoproxil fumarate (TDF; 5.1mg/kg), emtricitabine (FTC; 30mg/kg; both from Gilead), and dolutegravir (DTG; 2.5mg/kg; ViiV Healthcare), formulated in Kleptose (15% in 0.1 N NaOH, Roquette), a combination that was selected for its effectiveness in suppressing viral replication in RMs [43]. ART was started at week 8 p.i. to allow time for RMs to reach viral setpoint and establish late acute/early chronic SIVmac239 infection, as well as to model time to ART initiation in HIV/malaria co-endemic areas [44–48].

### *P. fragile* Inoculation, Monitoring, and Anti-Malarial Treatment

RMs were i.v. inoculated with 20×10^6^ *P. fragile*-infected erythrocytes (Sri Lanka strain) [49–51]. Clinical markers of *Plasmodium* infection were measured via complete blood counts (CBCs) and serum chemistries. Anemia was monitored via hematocrit (%HCT). Peripheral parasitemia was measured using Giemsa-stained thin blood smears [37]. Parasite detection was performed by qPCR as previously described [52, 53]. Briefly, DNA was extracted using a Quick-DNA/RNA Miniprep Kit (Zymo Research; whole blood) or a Quick-DNA Fecal/Soil Microbe Kit (Zymo Research; GI tissue). *P. fragile*-specific forward (5′-CAGCTTTTGATGTTACGGGTATTG-3′) and reverse (5′-CCTCTCCGGAATCGAACTCTAA-3′) primers, and a TaqMan *P. fragile*-specific probe (5′-CCTAACATGGCTATGACGGGTAACGGG-3′) (Integrated DNA Technologies; IDT), for the *P. fragile* 18S ribosomal subunit (GenBank M61722) were used [52, 53]. The PCR reaction was performed using 3-5µL of extracted DNA, 1µL of 300nM forward and reverse primer concentrations each, 1µl of 250nM probe concentrations, and 13µL of PrimeTime MasterMix (IDT) (20µl total volume per reaction), with the remaining volume made up of PCR-grade water (Thermo Fischer Scientific). Serially diluted *P. fragile* 18S DNA g-blocks (IDT) spiked with *P. fragile*-naïve RM genomic DNA were used as a standard curve, against which samples were quantified and reported as copies/µL. No-template and no-amplification negative controls were used in each reaction set.

Anti-malarial treatment was administered to RMs using the following guidelines: If % parasitemia rose above 0.5%, RMs were treated orally with a suboptimal dose of quinine sulfate (150mg daily; Archway Apothecary, NDC: 51927-1588-00) as previously described [37, 52, 53]. This allowed for the establishment of a chronic *Plasmodium* infection that models the prolonged exposure to *Plasmodium* that occurs in malaria-endemic areas [54, 55]. If parasitemia rose above 15%, RMs were switched to oral chloroquine treatment (20mg/kg; Health Warehouse, NDC: 64980-0177-50). Quinine sulfate and/or chloroquine treatments were halted when RMs were observed to be below the 0.5% parasitemia threshold and restarted if subsequent parasitemia levels again rose above 0.5% (Supplementary Table 1).

### Sample Collection and Processing

Peripheral blood was collected in EDTA and serum vacutainer tubes (Starstedt) via the femoral vein. CBCs and blood chemistry were performed as previously described [37]. EDTA blood was processed for plasma isolation and whole-blood flow cytometric analysis [37]. Up to 20 biopsies were endoscopically collected from the colon and duodenum using sterile biopsy forceps and allocated for 1) zinc-formalin fixation and paraffin embedding (FFPE) and 2) enzymatic digestion into single cell suspensions for flow cytometric staining and cryopreservation. Enzymatic digestions were performed by incubating with DNase and Liberase, followed by manual dissociation and distribution for flow cytometric analysis as previously described [56, 57].

### Flow Cytometry

Multi-color flow cytometric analysis was performed according to standard procedures and using monoclonal antibodies that cross-react with RMs [37]. Antibody information is listed in Supplementary Tables 2 and 3. Samples were stained with a Live/Dead Fixable Aqua dead cell stain (Thermo Fisher Scientific), then treated with Fc block (BD Biosciences) at room temperature. Extracellular staining was performed using predetermined fluorochrome conjugated antibody concentrations at 4°C (Supplementary Table 2 and 3), followed by red blood cell lysis using 1X FACS lysing solution (BD Biosciences) at room temperature. Cells were fixed, permeabilized (CytoFix/Perm Kit, BD Biosciences), then intracellularly stained (Supplementary Table 2 and 3) both at 4°C. Samples were fixed with 1% paraformaldehyde and held at 4°C until acquisition on a BD LSRFortessa using FACSDiva software (v9.0). Single-color controls were acquired in every experiment to enable compensation calculations. Analysis was performed using FlowJo (v10). In all analyses, a threshold of 100 events or more in the parental gate was required to report frequency. Representative gating strategies are shown in Supplementary Figures 1 and 2. Absolute CD4+ and CD8+ T cell counts were monitored by flow cytometry [37] (Supplementary Table 4).

### Histopathology

Sections from FFPE duodenum samples were cut, mounted, stained with hematoxylin and eosin (H&E), and analyzed by a veterinary pathologist for neutrophilic infiltration, hemozoin, hemosiderin, thrombi, epithelial degeneration, lymphoid hyperplasia, and infarcts. Slides were scanned on a NanoZoomer (Hamamatsu Photonics). Pathologists used the following system to score H&E lesions: 0=absent; 1+=minimal; 2+=mild; 3+=moderate; 4+=severe.

### Fluorescent Immunohistochemical Staining for Neutrophil Extracellular Traps

FFPE duodenum biopsies were sectioned at 4µm, mounted on Superfrost Plus Microscope slides (Fisher Scientific), and baked for 2 hours at 60°C. Slides were deparaffinized, rehydrated, and subsequently underwent heat-induced epitope retrieval, first in a Tris-based solution (pH 9.0) (H-3301; Vector Labs) then a citrate-based solution (pH 6.0) (H-3300; Vector Labs). Slides were washed in PBS then counterstained with DAPI (Supplementary Table 5). Slides underwent sequential rounds of blocking, primary and secondary antibody incubation, and color development on a Ventana Discovery Ultra automatic stainer (Roche; Supplementary Table 5). Between sequential staining rounds, slides underwent denaturation and neutralization to quench any remaining horse radish peroxidase (HRP). All slides underwent a second DAPI counterstain. Slides were permanently mounted and digitally imaged at 20X with a Zeiss Axio Scan.Z1 (Zeiss).

Analysis was performed by a pathologist using HALO HighPlex FL v4.2.14 (Indica Labs). Briefly, stained slides were imaged in 4 channels (405, 488, 568, 647), regions of interest were selected, and an algorithm for the detection of each marker was set based on fluorescent intensity thresholds. The proportion of granulocytes (myeloperoxidase, MPO+) that were forming NETS (citrullinated histone 3, CitH3+) was reported based on the number of CitH3+ cells among total granulocytes (MPO+CitH3- and MPO+CitH3+).

### *In situ* Hybridization and Immunohistochemistry for Detection of *P. fragile* and SIV in Duodenum

Detection of *P. fragile* was performed via *in situ* hybridization (ISH) (RNAscope; Advanced Cell Diagnostics) using FFPE duodenum tissues, as previously described [58]. Briefly, 4µm FFPE tissue sections were mounted, baked, deparaffinized, and allowed to dry at RT. An Advanced Cell Diagnostics RNAscope VS Universal Sample Prep kit and HRP Detection Reagents (Advanced Cell Diagnostics for both) were run on a Ventana Discovery Ultra automatic stainer. Tissue pretreatment included target retrieval at 97°C for 16 minutes, followed by protease treatment at 37°C for 16 minutes. Tissues were incubated with either a *P. fragile* (GenBank M61722) or DapB probe at 43°C for 2 hours (Supplementary Table 6). To ensure that the ISH fixation, processing, and staining methods used here did not significantly impact staining results, validation and optimization experiments were performed using negative and positive (heavily parasitized RM tissue) controls. The same positive control slide was included on all staining runs to ensure that ISH was performed without errors and that there were no significant batch effects between runs.

Following *in situ* hybridization, slides were transferred to PBS with 0.2% fish gelatin (PBS-FSG) placed in a humidified chamber, blocked with 10% normal goat serum (NGS) diluted in PBS-FSG for 40 minutes at room temperature. Slides were incubated for 60 minutes with a primary antibody against SIVmac251 gp41 (KK41) envelope protein (Supplementary Table 6). Slides were washed and incubated for 40 minutes with a secondary antibody (Supplementary Table 6). The slides were stained with DAPI and permanently mounted. Slides were digitally imaged at 20X magnification using an Axio Scan.Z1 slide scanner and analyzed using HALO (FISH v3.31 and HighPlex FL v4.2.14) for *P. fragile* and SIV, respectively. Given the small size of endoscopic biopsies used in this study, all tissue was included in the final analysis. The presence of *P. fragile* was reported as malaria probe/mm^2^ of analyzed tissue. The presence of SIV was reported as a density of KK41-positive cells/mm^2^ of analyzed tissue.

### Data and Statistical Analysis

For longitudinal clinical disease parameters, including *P. fragile* parasite copies and SIV loads detected by PCR, hematological parameters, and immune cell frequencies identified by flow cytometry and fluorescent immunohistochemistry, statistical significance between experimental time points and baseline was calculated using a mixed-effects analysis with the Geisser-Greenhouse correction and Dunnett's multiple comparisons test, with individual variances computed for each comparison. For some parameters, the area under the curve was calculated for ART-treated SIV/*P. fragile* co-infection timepoints (weeks 12–20). Correlations were performed using a 2-tailed, nonparametric Spearman's correlation. *P* values of < 0.05 were considered significant. All statistical analyses were performed using GraphPad Prism (Version 10).

## RESULTS

### Experimental Design

**Figure 1.**
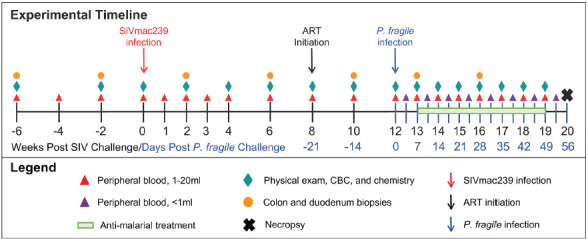
**Experimental timeline.** Experimental timeline depicting sample collection from adult male RMs (n=6). Time points in black font are shown relative to the weeks since SIVmac239 inoculation, while time points in blue font are shown relative to the days since *P. fragile* inoculation.

Following baseline sampling, RMs (n=6) were i.v. inoculated with SIVmac239 (Figure 1). For simplicity, all timepoints are stated as weeks relative to SIV inoculation. At week 8 post-SIV infection (p.i.), RMs initiated daily ART, delivered daily via subcutaneous injection until euthanasia at week 20 p.i. At week 12 p.i., all RMs were i.v. inoculated with *P. fragile*. RMs received anti-malarial drugs when parasitemia exceeded 0.5% beginning at week 13 through 18 p.i. (Supplementary Table 1). Physical exams, peripheral blood, and GI (colon and duodenum) biopsies were collected throughout the experimental timeline.

### Confirmation of Productive *P. fragile* and SIV Infection in RMs

**Figure 2.**
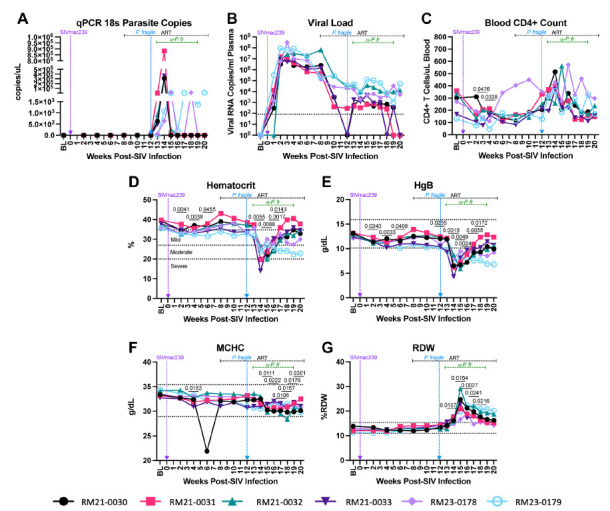
**Clinical parameters throughout ART-treated SIV*/P. fragile* co-infection in RMs*.*** Clinical parameters, complete blood counts (CBCs), and serum chemistries were monitored throughout ART-treated SIV/*P. fragile* co-infection (n=6). A) Parasitemia was quantified via quantitative polymerase chain reaction (qPCR) using primers and probes against the *P. fragile* 18S gene. B) Plasma viral load (RNA copies/mL plasma) was assessed by qPCR. C) Absolute number of CD4+ T cells per μL of blood was assessed via flow cytometry. D) Anemia was assessed by characterizing % hematocrit, defined as the ratio of red blood cells to total blood. E-G) Hemoglobin (HgB; E), mean corpuscular hemoglobin concentration (MCHC; F), and red blood cell distribution width (RDW; G) were assessed via CBCs and serum chemistry. In all panels, each individual RM is represented by a different symbol and color. Timepoints are connected by lines. Baseline (BL) is an average of data collected at weeks −6, −4, −2, and 0 p.i. Inoculation with SIVmac239 at week 0 p.i. is indicated by a vertical purple dashed arrow. Inoculation with *P. fragile* at week 12 p.i. is indicated by a vertical blue dashed arrow. Antiretroviral therapy (ART) was initiated at week 8 p.i. and is indicated by the horizontal black dashed line. Antimalarial administration occurred between weeks 13 and 19 and is indicated by the horizontal green dashed line. α-Pf = anti-malarial treatment. Dotted horizontal lines in E-H indicate the normal range observed in healthy RMs. Statistical significance at each time point compared to baseline was calculated using a mixed-effects analysis with the Geisser-Greenhouse correction and a Dunnett's multiple comparisons test, with individual variances computed for each comparison. Multiplicity-adjusted significant *P* values are shown above horizontal black bars.

Using qPCR, we found that 5/6 RMs had detectable copies of the *P. fragile* 18S gene at week 13 p.i., all RMs were detectable by week 14 p.i., and 4/6 RMs retained detectable levels at week 15 p.i. (Figure 2A). Two RMs had detectable *P. fragile* 18S copies until weeks 18 p.i. (RM23-0178) and 20 p.i. (RM23-0179) (Figure 2A). These findings were recapitulated in Giemsa staining of thin blood smears (Supplementary Figure 3). All RMs received at least one treatment with anti-malarial drugs between weeks 13 and 18 p.i. (Supplementary Table 1).

Following SIVmac239 inoculation, all RMs exhibited elevated viral loads that peaked by week 3 p.i. (median viral load=1.96×10^7^ copies/mL; Figure 1C). Following ART initiation, all RMs had decreased viral loads by week 10 p.i. (Figure 2B). After *P. fragile* inoculation, 6/6 RMs had persistently detectable viral loads through week 18 p.i. (Figure 2B). Despite no differences in daily ART treatment or timing/dose of *P. fragile* co-infection starting at week 18 p.i., 3/6 RMs exhibited transient SIV control, whereas the remaining 3/6 RMs maintained stable levels of viral burden (Figure 2B). Taken together, these data indicate that all RMs were productively infected with both pathogens, and RMs exhibited differences in post-ART viral control.

### Clinical Parameters of SIV and *P. fragile* Were Established During ART-Treated Co-Infection in RMs

We next examined the impact of *P. fragile* co-infection of ART-treated SIV-infected RMs on clinical parameters. All RMs had significantly lower peripheral absolute CD4+ T cell counts at weeks 3 and 4 p.i., as compared to baseline (*P* = 0.0476 and 0.0328, respectively), followed by a return to baseline levels after ART initiation (Figure 2C). During *P. fragile* co-infection, all RMs exhibited transient fluctuations in peripheral absolute CD4+ T cell counts, which returned to baseline or lower levels by week 20 p.i. (Figure 2C).

Anemia is a classic indicator of malaria severity [59, 60]. HIV can also induce mild anemia [61, 62]. Here, anemia, assessed via percent hematocrit (%HCT), or the ratio of red blood cells to total blood, was observed during SIV infection prior to ART initiation, with significant decreases in %HCT at weeks 2, 4, and 6 p.i., as compared to baseline (*P* = 0.0041, 0.0038, and 0.0455, respectively; Figure 2D). Following *P. fragile* co-infection, %HCT was significantly decreased at weeks 14, 15, 16, and 17 p.i. as compared to baseline (*P* = 0.0055, 0.0088, 0.0017, and 0.143, respectively; Figure 2D).

Prior work has shown that serum levels of hemoglobin (HgB) are also decreased in both severe malaria and HIV infection [62–65]. Here, HgB was significantly decreased during acute SIV infection at weeks 2, 4, and 6 p.i. (*P =* 0.0240, 0.0033, and 0.0408, respectively), and following 1 month of ART (week 12 p.i.; *P =* 0.0255), as compared to baseline (Figure 2E). Following *P. fragile* co-infection, HgB was significantly decreased at weeks 14, 15, 16, 17, and 18 p.i., as compared to baseline (*P = 0*.0019, 0.0049, 0.0024, 0.0058, and 0.0172, respectively; Figure 2E). Notably, both %HCT and HgB values fell below the normal reference ranges in RMs after *P. fragile* co-infection.

Decreased blood and serum mean corpuscular hemoglobin concentration (MCHC) and increased red blood cell distribution width (RDW) have been observed in severe malaria [63–65]. Here, MCHC was significantly decreased at weeks 4, 15, 16, 17, 18, 19, and 20 p.i., as compared to baseline (*P =* 0.0153, 0.0111, 0.0222, 0.0106, 0.0157, 0.0176, and 0.0301, respectively; Figure 2F). RDW was significantly increased at weeks 14, 15, 16, 17, and 18 p.i., as compared to baseline (*P* = 0.0157, 0.0154, 0.0027, 0.0241, and 0.0216, respectively; Figure 2G). Taken together, these data indicate that clinical parameters followed patterns expected from both infections and that co-infection exacerbated disruptions in hematological morphology.

### *P. fragile* Co-Infection of ART-Treated SIV-Infected RMs Does Not Impact CD4+ T Cell Restoration

**Figure 3.**
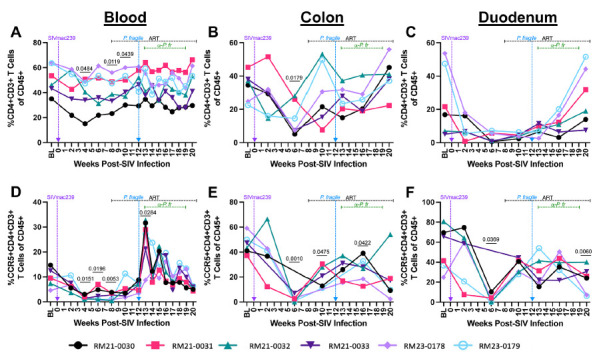
**T cell subset frequencies throughout ART-treated SIV/*P. fragile* co-infection in RMs.** Total CD4+ T cell frequencies and CCR5+ CD4+ T cell frequencies were assessed in whole blood and colon and duodenum biopsies throughout ART-treated SIV/*P. fragile* co-infection via flow cytometry (n=6). A-C) The frequency of CD4+ T cells (CD3+CD4+) of viable CD45+ cells was assessed in whole blood (A), colon (B), and duodenum (C). D-F) The frequency of CCR5+ CD4+ T cells of viable CD45+ cells was assessed in whole blood (D), colon (E), and duodenum (F). In all panels, each individual RM is represented by a different symbol and color. Timepoints are connected by lines. Baseline (BL) is an average of data collected at weeks −6, −4, −2, and 0 p.i. Inoculation with SIVmac239 at week 0 p.i. is indicated by a vertical purple dashed arrow. Inoculation with *P. fragile* at week 12 p.i. is indicated by a vertical blue dashed arrow. Antiretroviral therapy (ART) was initiated at week 8 p.i. and is indicated by the horizontal black dashed line. Antimalarial administration occurred between weeks 13 and 19 and is indicated by the horizontal green dashed line. α-Pf = anti-malarial treatment. Statistical significance at each time point compared to baseline was calculated using a mixed-effects analysis with the Geisser-Greenhouse correction and a Dunnett's multiple comparisons test, with individual variances computed for each comparison. Multiplicity-adjusted significant *P* values are shown above horizontal black bars.

The impact of *Plasmodium* spp. co-infection on HIV/SIV-associated CD4+ T cell depletion is not well defined, particularly in the GI mucosa. Therefore, we next examined peripheral and GI mucosal CD4+ T cell frequencies during ART-treated SIV/*P. fragile* co-infection. As expected, RMs exhibited a significant reduction in peripheral CD4+ T cell frequencies at weeks 4, 8, and 10 p.i. (*P =* 0.0484, 0.0119, and 0.0439, respectively; Figure 3A), as compared to baseline. In the colon, RMs had significantly lower CD4+ T cell frequencies at week 10 p.i. (*P* = 0.0179), followed by a return to baseline levels after ART initiation (Figure 3B). Although not statistically significant, CD4+ T cell frequencies were reduced in the duodenum during SIV infection, which increased to baseline levels after ART (Figure 3B). Notably, peripheral, colon, and duodenum CD4+ T cell frequencies were unchanged during *P. fragile* co-infection of ART-treated SIV-infected RMs, as compared to baseline (Figure 3A-C).

### CCR5+ CD4+ T cells Remain Persistently Depleted in the Colon and Duodenum During *P. fragile* Co-Infection of ART-Treated SIV-Infected RMs

CCR5+ CD4+ T cells are a major target population of HIV/SIV that are persistently depleted in the GI mucosa [66]. Here, we observed that this subset was significantly reduced in the periphery at weeks 4, 6, and 8 p.i. (*P* = 0.0151, 0.0196, and 0.0053, respectively), as compared to baseline (Figure 3D). At week 13 p.i., which coincided with peak *P. fragile* parasitemia, all RMs experienced significant expansion of peripheral CCR5+ CD4+ T cells (*P* = 0.0284), followed by a decline in the frequency of this population after anti-malarial treatment and parasite clearance (Figure 3D). As compared to baseline, CCR5+ CD4+ T cell frequencies in the colon were significantly depleted at week 6 p.i. (*P =* 0.0010), continued to be significantly reduced after 2 weeks of ART (week 10 p.i., *P* = 0.0475), and remained significantly lower following *P. fragile* co-infection (week 16 p.i., *P* = 0.0422; Figure 3E). Likewise, CCR5+ CD4+ T cell frequencies in the duodenum were significantly reduced at week 6 p.i. (*P* = 0.0422), partially recovered following ART initiation, but were significantly lower following *P. fragile* co-infection, antimalarial treatment, and parasite clearance (week 20 p.i., *P* = 0.0060), as compared to baseline (Figure 3F). Taken together, these findings suggest that RMs experienced classic CCR5+ CD4+ T cell depletion in the periphery and GI tract during acute SIV infection and that this subset remains persistently depleted in the GI mucosa throughout *P. fragile* co-infection.

### Minimal Alterations in Mucosal Neutrophil Frequencies Following *P. fragile* Co-Infection of ART-Treated SIV-Infected RMs

**Figure 4.**
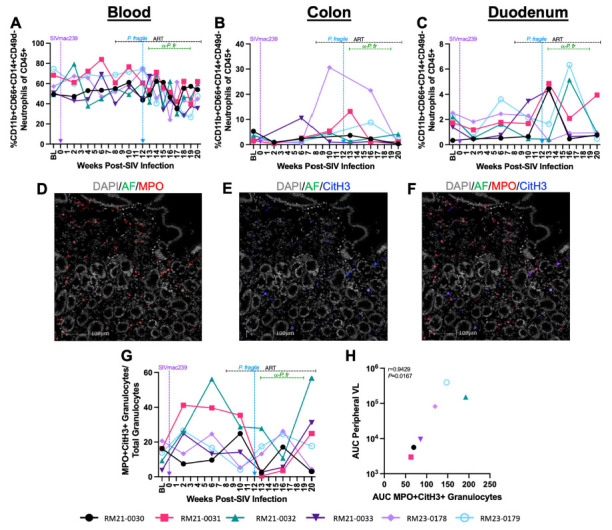
**Neutrophil and NET-forming granulocyte frequencies throughout ART-treated SIV/*P. fragile* co-infection in RMs.** Total neutrophil frequencies were assessed in whole blood and colon and duodenum biopsies, and NET-forming granulocyte frequencies were assessed in duodenum biopsies throughout ART-treated SIV/*P. fragile* co-infection in RMs via flow cytometry and fluorescent immunohistochemistry (n=6). A-C) The frequency of neutrophils (CD11b+CD66abce+CD14+CD49d-) of viable CD45+ cells was assessed in whole blood (A), colon (B), and duodenum (C). D-F) Representative images of duodenum biopsies collected at week 13 p.i. stained for myeloperoxidase (MPO, red; D) and citrullinated histone 3 (CitH3, blue; E). NET-forming granulocytes were identified as MPO+CitH3+ (F). In D-F, autofluorescence (AF) = green. DNA/DAPI = white. Bar = 100μm. G) Proportion of MPO+CitH3+ granulocytes among total granulocytes identified in the duodenum. H) Area under the curve (AUC) of NET-forming granulocyte-frequencies in the duodenum following *P. fragile* co-infection was correlated with the peripheral viral load AUC following *P. fragile* co-infection (weeks 12–20 p.i.) using a Spearman's test. For A-C and G-H, each individual RM is represented by a different symbol and color. In A-C and G, timepoints are connected by lines. Baseline (BL) is an average of data collected at weeks −6, −4, −2, and 0 p.i. Inoculation with SIVmac239 at week 0 p.i. is indicated by a vertical purple dashed arrow. Inoculation with *P. fragile* at week 12 p.i. is indicated by a vertical blue dashed arrow. Antiretroviral therapy (ART) was initiated at week 8 p.i. and is indicated by the horizontal black dashed line. Antimalarial administration occurred between weeks 13 and 19 and is indicated by the horizontal green dashed line. α-Pf = anti-malarial treatment. Statistical significance at each timepoint compared to baseline was calculated using a mixed-effects analysis with the Geisser-Greenhouse correction and a Dunnett's multiple comparisons test, with individual variances computed for each comparison.

Our prior work showed that elevated peripheral frequencies of activated CD4+ and CD8+ T cells were significantly positively correlated with inflammatory neutrophil frequencies during acute SIV and early ART [67]. Therefore, we next characterized neutrophil frequency in the periphery and directly in the GI mucosa with the goal of identifying whether alterations in this innate immune subset could drive elevations in clinical parameters and contribute to disruptions in CD4+ T cell subsets during ART-treated SIV/*P. fragile* co-infection. Peripheral neutrophil (CD45+CD-11b+CD66abce+CD14+CD49d-) frequencies were unchanged as compared to baseline during acute SIV infection and in the first month following ART initiation (Figure 4A).

Following *P. fragile* co-infection, neutrophil frequencies declined below baseline levels, although this loss did not reach statistical significance (Figure 4A). In the colon, no significant differences in neutrophil frequencies were observed over time (Figure 4B). Similarly, neutrophil frequencies were unchanged in the duodenum during acute SIV and ART, although transient, non-significant fluctuations were observed following *P. fragile* co-infection and antimalarial treatment (Figure 4C). As no significant alterations in neutrophil frequencies were observed, we further examined the frequencies of dendritic cells, monocytes, macrophages, and natural killer cells in the periphery, colon, and duodenum; however, these subsets were also unchanged throughout co-infection (Supplementary Figure 4). Taken together, ART-treated SIV/*P. fragile* co-infection did not result in alterations in peripheral and mucosal innate immune cell frequencies, including neutrophils.

### NET Formation in the Duodenum of ART-Treated SIV/*P. fragile* Co-Infected Correlates With Peripheral Viremia

Although we did not observe differences in neutrophil frequency, it is possible that changes in mucosal neutrophil function could contribute to exacerbated SIV and *P. fragile* pathogenesis. Therefore, we next sought to identify whether NET formation in the mucosa during ART-treated SIV*/P. fragile* co-infection could contribute to clinical manifestations of SIV and *P. fragile* and GI immune dysfunction. Here, we specifically focused on the duodenum, as sustained CD4+ T cell depletion, including CCR5+ CD4+ T cell loss, and transient fluctuations in neutrophil frequency were observed in this tissue. Using fluorescent immunohistochemistry to identify NET-forming (citrullinated histone 3, CitH3+) granulocytes (MPO+) in duodenum tissue (Figure 4D-F), we observed non-significant increases in NET-forming granulocyte frequencies in the duodenum during acute SIV, which normalized following ART and were unchanged during *P. fragile* co-infection and antimalarial treatment (Figure 4G).

Next, we sought to determine whether duodenum NET formation was linked with clinical manifestations of ART-treated SIV and *P. fragile* following co-infection. To do this, we first calculated the area under the curve during co-infection (weeks 12-20) for parameters including NET-forming granulocyte frequency, peripheral viral load, parasitemia, CD4+ and CCR5+ CD4+ T cell frequency, and duodenum CD4+ and CCR5+ CD4+ T cell frequency. We observed a significant correlation between NET-forming granulocyte frequency and peripheral viral load following co-infection (r = 0.9429, *P* = 0.0167; Figure 4H). However, NET-forming granulocyte frequency during co-infection was not significantly correlated with peripheral parasitemia (r = −0.5429, *P* = 0.2972), CD4+ or CCR5+ CD4+ T cell frequency (r = −0.08571, *P* = 0.9194 and r = 0.6, *P* = 0.2417, respectively) or duodenum CD4+ or CCR5+ CD4+ T cell frequency (r = 0.3714, *P* = .4972 and r = 0.3413, *P* = 0.5639, respectively). Taken together, these data indicate that duodenum NET formation may be linked with peripheral viremia but not peripheral parasitemia nor peripheral or mucosal CD4+ T cell dynamics during ART-treated SIV/*P. fragile* co-infection.

### *P. fragile* Parasite Burden in the Duodenum is Not Linked With NET-Forming Granulocyte Levels During ART-Treated SIV Infection

**Figure 5.**
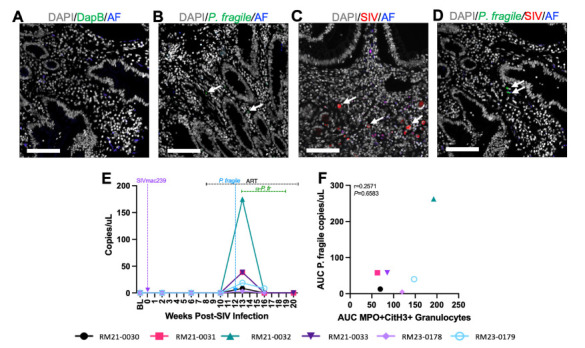
**Detection of SIV and *P. fragile* in the duodenum during ART-treated SIV/*P. fragile* co-infection in RMs.** The presence of *P. fragile* and SIV in duodenum biopsies throughout ART-treated SIV/*P. fragile* co-infection was assessed via *in situ* hybridization and fluorescent immunohistochemistry, respectively (n=6). A) Duodenum tissue from RM21-033, stained with the negative control probe for DapB (green), showed no signal. B) RM22-0116, an RM infected with *P. fragile* only, had positive staining for the *P. fragile* 18S gene (green, arrows) in the lamina propria of the duodenum at necropsy. C) RM25-0254, an RM infected with SIVmac239 only, had positive staining for KK41 (SIV env, red, arrows) in the lamina propria of the duodenum at necropsy. D) RM21-033 had positive staining for the *P. fragile* 18S gene (green arrows) in the lamina propria of duodenum biopsies. In A-D, autofluorescence (AF) = blue. DNA/DAPI = white. Bar = 100μm. E) 18S *P. fragile* parasite copies (copies/μL) were quantified and calculated using quantitative polymerase chain reaction (qPCR). H) Area under the curve (AUC) of NET-forming granulocyte-frequencies in the duodenum following *P. fragile* co-infection was correlated with the AUC of *P. fragile* copies in the duodenum following *P. fragile* co-infection (weeks 12-20 p.i.) using a Spearman's test. For E-F, each individual RM is represented by a different symbol and color. In E, timepoints are connected by lines. Baseline (BL) is an average of data collected at weeks −6, −4, −2, and 0 p.i. Inoculation with SIVmac239 at week 0 p.i. is indicated by a vertical purple dashed arrow. Inoculation with *P. fragile* at week 12 p.i. is indicated by a vertical blue dashed arrow. Antiretroviral therapy (ART) was initiated at week 8 p.i. and is indicated by the horizontal black dashed line. Antimalarial administration occurred between weeks 13 and 19 and is indicated by the horizontal green dashed line. α-Pf = anti-malarial treatment. Statistical significance at each timepoint compared to baseline was calculated using a mixed-effects analysis with the Geisser-Greenhouse correction and a Dunnett's multiple comparisons test, with individual variances computed for each comparison.

Given the association between NET-forming granulocyte levels and peripheral viral load during ART-treated SIV/*P. fragile* co-infection, and previous studies demonstrating *P. falciparum* sequestration in the GI tract [29–31], we next characterized whether *P. fragile* burden in the duodenum could drive NET formation and subsequently influence peripheral SIV viral load. We first used *in situ* hybridization (ISH) and IHC to identify the presence of *P. fragile* and SIV in the duodenum (Figure 5A-D). We observed that *P. fragile* parasites, but not SIV-infected cells, were present in the duodenum at week 13 p.i. (Figure 5D). Quantification of *P. fragile* parasites via qPCR revealed that the greatest *P. fragile* burden in the duodenum was at week 13 p.i., which coincided with peak peripheral *P. fragile* parasitemia (Figure 5E).

No association was observed between total *P. fragile* parasite burden during co-infection, calculated as the area under the curve of duodenum *P. fragile* levels between weeks 12 and 20 p.i., and total NET-forming granulocyte levels during co-infection (r = 0.2571, *P* = 0.6583; Figure 5F). Moreover, histopathological analysis of duodenum tissue revealed no significant differences in mucosal damage over time (Supplementary Figure 5). Taken together, although *P. fragile* parasites were observed in the GI tract during ART-treated SIV co-infection, they were not linked with NET-forming granulocyte frequency or histopathological evidence of mucosal damage, indicating that *P. fragile*/neutrophil interactions in the GI tract may not contribute to peripheral viremia or GI damage.

## DISCUSSION

Here, we used the RM model to characterize the impact of ART-treated SIV/*P. fragile* co-infection on the GI mucosa. As expected, all RMs exhibited peripheral viremia and depleted CD4+ T cell counts during acute SIV infection, which were partially restored by ART. Moreover, consistent with prior reports, all RMs exhibited peripheral parasitemia following *P. fragile* inoculation [37, 52, 53], which coincided with increased RDW and decreased %HCT, HgB, and MCHC [3, 68–70]. Taken together, these data demonstrate that RMs were productively infected with both SIV and *P. fragile* and that both infections followed expected kinetics.

The massive and persistent depletion of CD4+ T cells from the GI mucosa during HIV/SIV infection and incomplete restoration of this immune compartment with ART has been well documented [25, 71, 72] and contributes to the mucosal immune dysfunction characteristic of pathogenic HIV/SIV infection. In addition, prior work has shown that CD4+ T cells expressing the HIV/SIV co-receptor CCR5 are almost completely lost in mucosal tissue during HIV and SIV infection [66, 73]. Here, we observed a significant expansion of CCR5+ CD4+ T cells in the periphery following *P. fragile* co-infection of ART-treated SIV-infected RMs. This is consistent with previous studies, which demonstrated that infection of SIV-naïve RMs with *P. cynomolgi* resulted in expansion of CCR5+ CD4+ T cells [74, 75].

A notable finding in our study was that *P. fragile* co-infection following ART-treated SIV infection did not appear to impact total CD4+ T cell restoration in the periphery or GI mucosa following ART, or persistent CCR5+ CD4+ T cell depletion in the GI mucosa despite ART. This is consistent with prior work demonstrating that RMs infected with SIVmac251 followed by co-infection with *P. cynomolgi* did not exhibit differences in the frequency of blood CCR5+ CD4+ T cells as compared to RMs with SIVmac251 only [74]. Thus, our findings represent an important extension of previous work on SIV/*Plasmodium* co-infection into a critical tissue site that is significantly impacted during HIV/SIV infection. Further, our data indicate that the transient expansion of CCR5+ CD4+ T cells in the periphery may not only be a consequence of ART but an additional *P. fragile*-specific T cell response and that *Plasmodium* co-infection may not impact CCR5+ CD4+ T cell recovery in the mucosa even in the context of ART.

Neutrophils are a critical component of host innate immunity [76–78]. However, dysregulated neutrophil activation can cause collateral host damage via uncontrolled inflammation, which is elevated during both HIV and malaria [31, 33, 35]. NETs capture and eliminate invading pathogens, including HIV and *Plasmodium*-infected red blood cells [79–82]. However, excessive NET formation results in increased inflammation, collateral host-tissue damage, and adverse HIV and *Plasmodium* clinical outcomes [83–87]. We recently showed that peripheral neutrophil frequency correlated with CD4+ T cell activation during acute SIV infection and early ART [67]. Therefore, we expanded upon our prior studies to examine neutrophil dynamics directly in the GI mucosa during ART-treated SIV/*P. fragile* co-infection.

A notable finding here was that the frequency of NET-forming granulocytes in the duodenum during ART-treated SIV/*P. fragile* co-infection was positively associated with peripheral viral load during co-infection. These data may indicate that *P. fragile*-induced elevations in NET formation in the duodenum could result in GI immune activation, which may, in turn, drive peripheral viral replication in other sites. However, our data also indicated that duodenum *P. fragile* burden was not associated with levels of NET formation, suggesting that the mere presence of parasites in mucosal tissue alone does not induce NET formation.

Our findings are interesting in light of our previous work, which indicated that alterations in circulating markers of NET formation were associated with increased clinical signs of both SIV and *P. fragile,* increased peripheral markers of inflammation, and elevated plasma levels of GI permeability and microbial translocation [37]. It is possible that parasite sequestration in anatomical sites other than the GI tract could have contributed to the elevated levels of circulating markers of neutrophil activation and NET formation that we observed previously.

Additionally, it is possible that alternative mechanisms of GI dysfunction, such as GI microbial dysbiosis, could synergically combine with GI NET-formation to drive increases in GI permeability and microbial translocation. However, further investigation is required to confirm these theories and fully examine the mechanisms by which they may occur. In sum, changes in mucosal GI neutrophil function during ART-treated SIV/*P. fragile* co-infection could augment GI immune disruptions and subsequently contribute to persistent peripheral SIV replication. Further work, including longitudinal assessments of cell-associated viral DNA and RNA in blood and tissues, is needed to comprehensively determine the impact of *P. fragile* co-infection on total viral burden and replication in the context of ART.

A major strength of our study was the utilization of an established model of ART-treated SIV/*P. fragile* co-infection that mimics key aspects of HIV and *P. falciparum* infection, separately and during co-infection [32, 49–51]. Additionally, our study design enabled longitudinal monitoring and tissue sampling not feasible in other translational models of HIV and malaria.

A limitation of our study was that RMs were on ART for a short period of time, which did not allow for complete viral suppression prior to co-infection with *P. fragile*. Our rationale for initiating ART at week 8 p.i. was to allow RMs to establish an early viremic setpoint, reflective of late acute/early chronic SIV infection, which can occur as early as 42 days p.i. [44–46]. Although ART can reduce viremia within 2 weeks, full viral suppression may take longer [43]. In humans, despite guidelines recommending immediate ART initiation, delays in both diagnosis and treatment initiation are common [47, 48]. Thus, even with rapid testing and same-day ART initiation, early viral pathogenesis is often underway by the time treatment begins. Considering data from both humans and non-human primates (NHPs), we elected to initiate ART at week 8 p.i. to balance the goals of modeling early diagnosis and treatment while ensuring that pathogenic SIV infection was established prior to ART. We acknowledge that this study design limits the conclusions that can be made regarding the impact of *P. fragile* co-infection during fully suppressed SIV. Future studies should address these limitations by extending the time on ART to allow for full viral suppression prior to co-infection.

An additional limitation of our study is that we examined the effects of a single exposure to *P. fragile* in ART-treated SIV-infected RMs, which does not account for the possibility of previous exposures or the potential for reinfection following parasite clearance. In malaria-endemic areas, recurrent *Plasmodium* spp. infections are common [88] and may occur both before and after HIV infection. Thus, future studies that examine the impact of *P. fragile* exposure before and after SIV and/or ART are needed. Finally, an inherent limitation of this study was the small sample size, which could explain inter-animal variation and lack of statistical significance in some parameters at certain time points. Moreover, inclusion of SIV- or *P. fragile*-only control groups was not possible within this study, so our assessments were limited to within-animal changes over time. Additional work with larger sample sizes and appropriate single-infection control groups is warranted.

## CONCLUSION

In summary, the data presented here suggest that although *P. fragile* co-infection during ART-treated SIV infection does not impact peripheral and mucosal CD4+ T cell and neutrophil frequencies, NET-forming granulocytes that occur in the GI tract during *P. fragile* infection may be linked with peripheral SIV replication despite ART. These data may have implications for HIV and malaria in co-endemic settings. Namely, neutrophil-derived inflammation is a potential mechanism that may promote viral replication in *Plasmodium* spp. co-infected PWH on ART. Future studies are needed to fully confirm the mechanism by which inflammatory processes, particularly in the GI tract, could contribute to adverse outcomes during ART-treated HIV/*Plasmodium* co-infection. Taken together, our work specifically highlights the need to continue defining the impacts of HIV and *Plasmodium* spp., separately and in the context of co-infection and ART, on immune function with the goal of developing immune-targeting therapeutics and strategies to enhance protection against both infections.

## Data Availability

All data are available within the manuscript or in the supplementary materials.

## SUPPLEMENTARY DATA

Supplementary materials are available at the *Pathogens and Immunity* website. Supplementary data may be provided by the authors to benefit the reader. Supplementary data are not copyedited and are the sole responsibility of the authors. Questions or comments related to supplementary materials should be addressed to the corresponding author.
