# Supplementary material for: Gastrointestinal Mucosal Disruptions During ART-Treated SIV/Plasmodium fragile Co-Infection
Source: Pathog Immun. 2026 Feb 3;11(1):39–65. doi: 10.20411/pai.v11i1.854 (PMC12880234; doi:10.20411/pai.v11i1.854)
Supplement: Supplementary Tables and Figures [file pai-11-039-s01.pdf]

PATHOGENS AND IMMUNITY
IN ASSOCIATION WITH CASE WESTERN RESERVE UNIVERSITY AND THE RICHARD J. FASENMYER FOUNDATION

Supplementary Figure 1

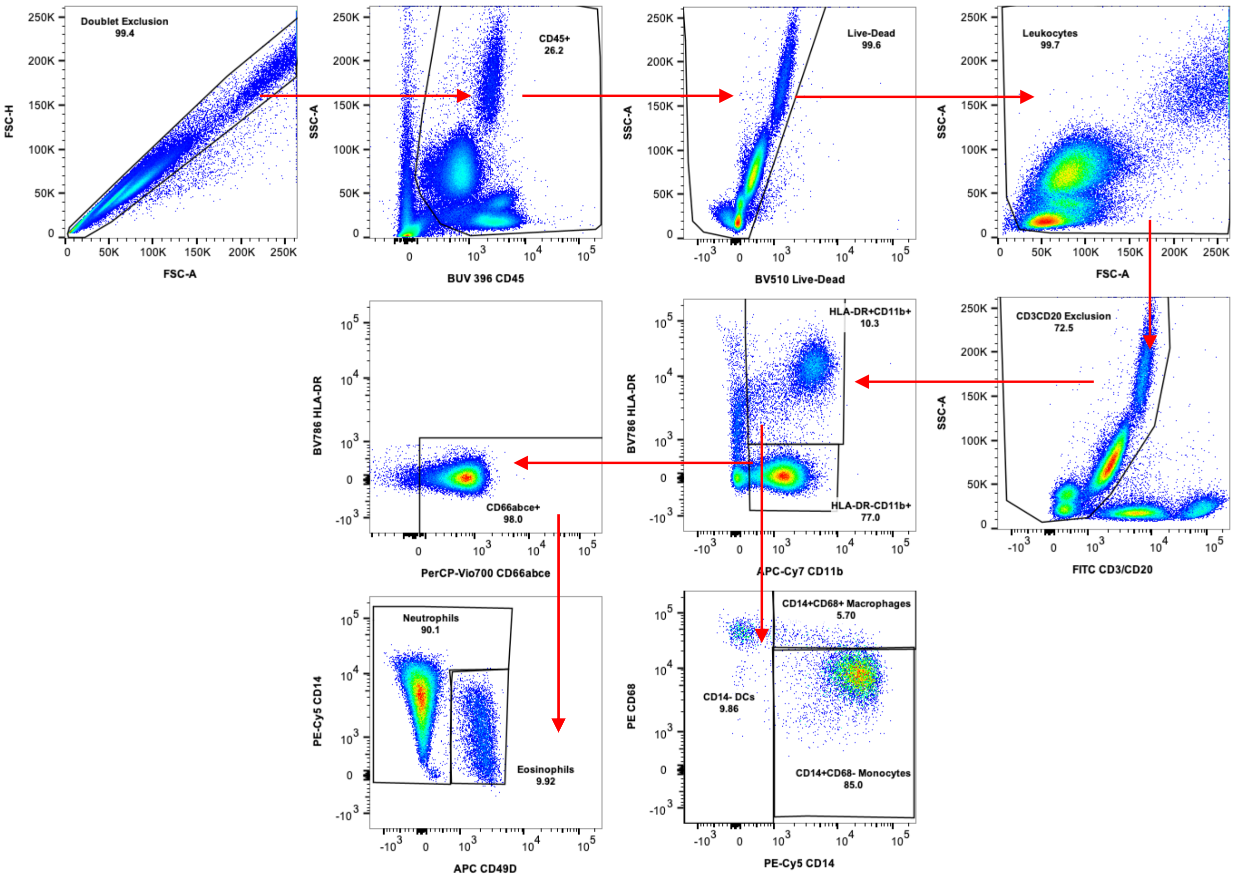

Representative flow plots demonstrating gating strategy used to identify and phenotype myeloid cells. Multicolor flow cytometry was used to identify myeloid cells in whole blood and colon and duodenum biopsies. Depicted here are representative plots from the whole blood of a rhesus macaque (RM21-030) prior to SIVmac239 inoculation (week -2). Doublets were first excluded using forward scatter (FSC) area and height (FSC-A and FSC-H, respectively) properties. Next, CD45+ cells were identified, dead cells were excluded using an Aqua Live/Dead viability dye, and remaining debris was removed using FSC and side scatter (SSC) properties. T cells and B cells were excluded by gating on CD3- and CD20- cells. Granulocytes were identified as HLA-DR-CD11b+ cells. Among total granulocytes, neutrophils and eosinophils were identified as CD66abce+ cells and then further classified as neutrophils (CD14+CD49d-) and eosinophils (CD14dimCD49d+). Monocytes, macrophages, and DCs were identified as HLA-DR+CD11b+ cells. Macrophages were further identified as CD14+CD68+, monocytes as CD14+CD68-, and DCs as CD14-.

Nemphos SM, Green HC, Prusak JE, Fell SL, Midkiff C, Rodgers A, Perret J, Goff K, Miller J, Varnado M, Didier K, Valencia N, Moström MJ, Tatum C, Barnes MB, Krzykwa CE, Rowe LA, Allers C, Grasperge B, De Paris K, Maness NJ, Kaur A, Londono-Renteria B, Blair RV, Manuzak JA. Gastrointestinal Mucosal Disruptions During ART-Treated SIV/*Plasmodium fragile* Co-Infection. *Pathogens and Immunity*. 2026;11(1):39–65. doi: 10.20411/pai.v11i1.854

PATHOGENS AND IMMUNITY
IN ASSOCIATION WITH CASE WESTERN RESERVE UNIVERSITY AND THE RICHARD J. FASENMYER FOUNDATION

Supplementary Figure 2

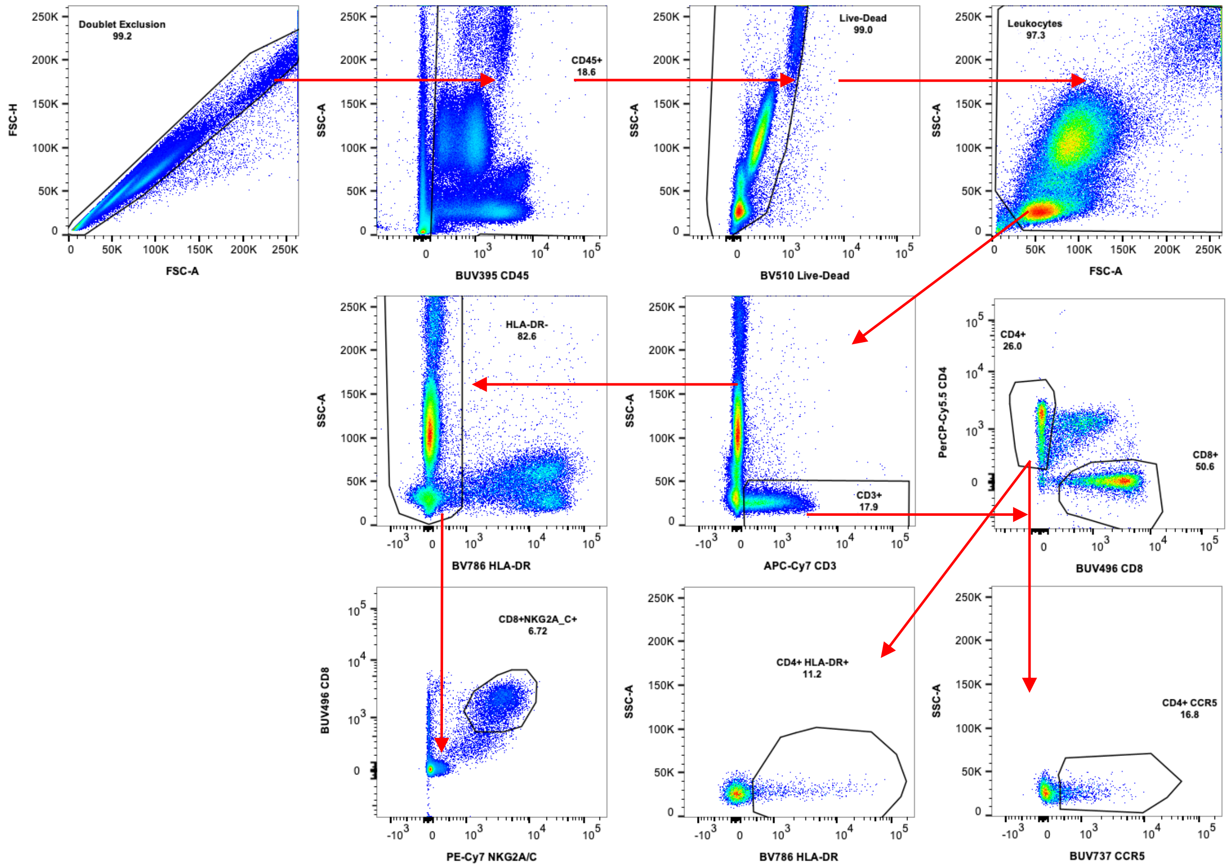

Representative flow plots demonstrating gating strategy used to identify and phenotype lymphoid cells. Multicolor flow cytometry was used to identify lymphoid-derived cells in whole blood and colon and duodenum biopsies. Depicted here are representative plots from the whole blood of a rhesus macaque (RM21-030) prior to SIVmac239 inoculation (week -2). Doublets were first excluded using forward scatter (FSC) area and height (FSC-A and FSC-H, respectively) properties. Next, CD45+ cells were identified, dead cells were excluded using an Aqua Live/Dead viability dye, and remaining debris was removed using FSC and side scatter (SSC) properties. Next, T cells were identified by CD3+ expression against SSC and were further delineated by either CD4+ or CD8+ expression. CD4+ T cells were further delineated by HLA-DR+ or CCR5+ expression. All CD3- cells were gated for negative expression of HLA-DR. Natural killer (NK) cells were identified from HLA-DR- cells and defined as CD8+NKG2A/C+.

Nemphos SM, Green HC, Prusak JE, Fell SL, Midkiff C, Rodgers A, Perret J, Goff K, Miller J, Varnado M, Didier K, Valencia N, Moström MJ, Tatum C, Barnes MB, Krzykwa CE, Rowe LA, Allers C, Grasperge B, De Paris K, Maness NJ, Kaur A, Londono-Renteria B, Blair RV, Manuzak JA. Gastrointestinal Mucosal Disruptions During ART-Treated SIV/*Plasmodium fragile* Co-Infection. *Pathogens and Immunity*. 2026;11(1):39–65. doi: 10.20411/pai.v11i1.854

PATHOGENS AND IMMUNITY
IN ASSOCIATION WITH CASE WESTERN RESERVE UNIVERSITY AND THE RICHARD J. FASENMYER FOUNDATION

Supplementary Figure 3

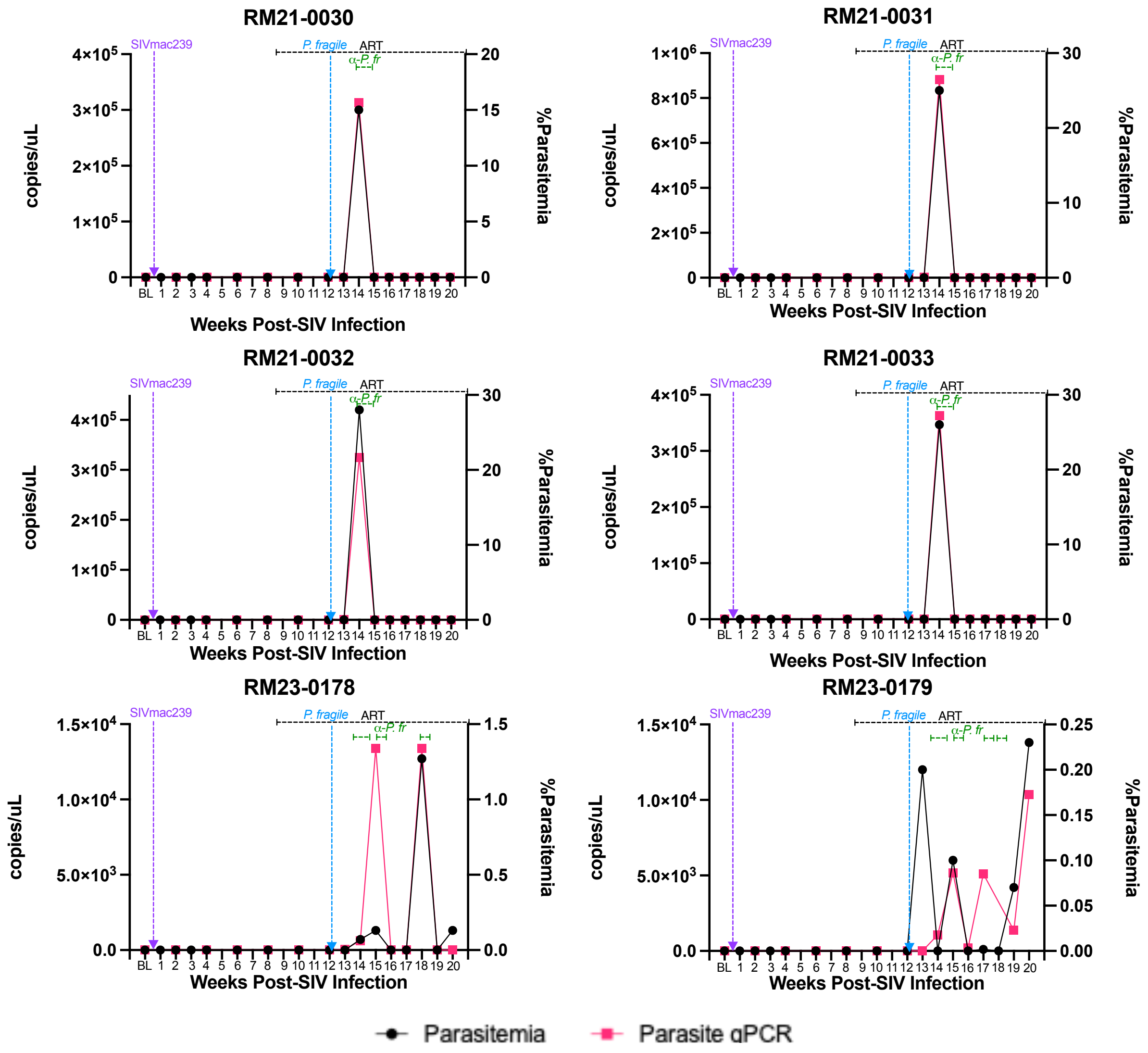

Peripheral parasitemia by Giemsa staining compared to qPCR quantification of *P. fragile* 18S rRNA gene. % Parasitemia was assessed via Giemsa staining of thin blood smears and was defined as the percentage of erythrocytes infected by a parasite among all erythrocytes. Parasite copies/µl were quantified via quantitative polymerase chain reaction (qPCR) using *P. fragile*-specific primers and probes against the *P. fragile* 18S gene. Parasitemia and qPCR are represented by different colors. Baseline (BL) is an average of data collected at weeks -6, -4, -2, and 0 p.i. Inoculation with SIVmac239 at week 0 p.i. is indicated by a vertical purple dashed arrow. Inoculation with *P. fragile* at week 12 p.i. is indicated by a vertical blue dashed arrow. Antiretroviral therapy (ART) was initiated at week 8 p.i. and is indicated by the horizontal black dashed line. Antimalarial administration occurred between weeks 13 and 19 and is indicated by the horizontal green dashed line. α-Pf = anti-malarial treatment.

Nemphos SM, Green HC, Prusak JE, Fell SL, Midkiff C, Rodgers A, Perret J, Goff K, Miller J, Varnado M, Didier K, Valencia N, Moström MJ, Tatum C, Barnes MB, Krzykwa CE, Rowe LA, Allers C, Grasperge B, De Paris K, Maness NJ, Kaur A, Londono-Renteria B, Blair RV, Manuzak JA. Gastrointestinal Mucosal Disruptions During ART-Treated SIV/*Plasmodium fragile* Co-Infection. *Pathogens and Immunity*. 2026;11(1):39–65. doi: 10.20411/pai.v11i1.854

Supplementary Figure 4

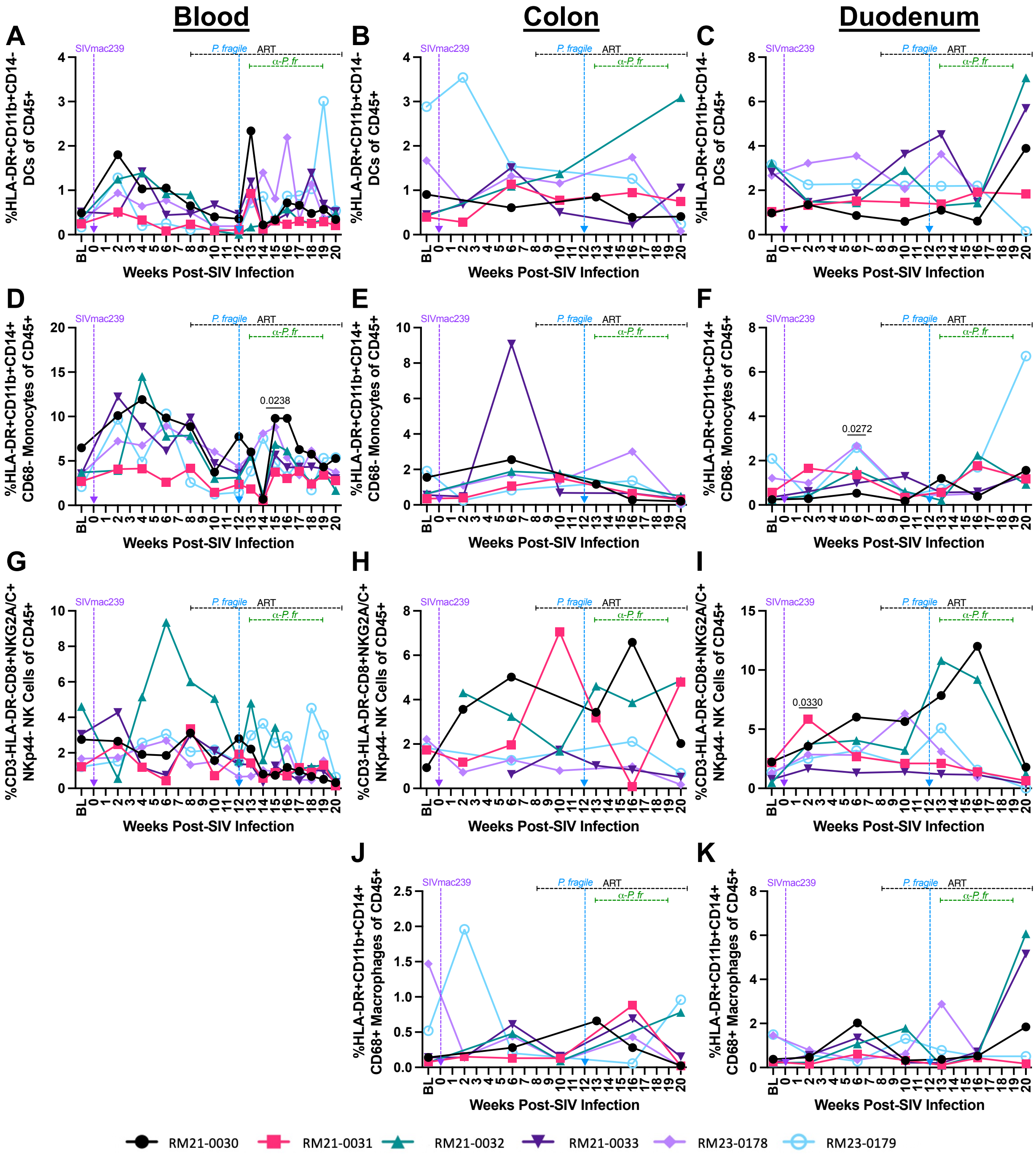

Innate immune cell subsets frequencies throughout ART-treated SIV/*P. fragile* co-infection in RMs. Immune cell frequencies were assessed in whole blood and colon and duodenum biopsies throughout ART-treated SIV/*P.*

Nemphos SM, Green HC, Prusak JE, Fell SL, Midkiff C, Rodgers A, Perret J, Goff K, Miller J, Varnado M, Didier K, Valencia N, Moström MJ, Tatum C, Barnes MB, Krzykwa CE, Rowe LA, Allers C, Grasperge B, De Paris K, Maness NJ, Kaur A, Londono-Renteria B, Blair RV, Manuzak JA. Gastrointestinal Mucosal Disruptions During ART-Treated SIV/*Plasmodium fragile* Co-Infection. *Pathogens and Immunity*. 2026;11(1):39–65. doi: 10.20411/pai.v11i1.854

PATHOGENS AND IMMUNITY
IN ASSOCIATION WITH CASE WESTERN RESERVE UNIVERSITY AND THE RICHARD J. FASENMYER FOUNDATION

fragile co-infection via flow cytometry (n=6). A-C) Dendritic cells were defined as HLA-DR+CD11b+CD14- cells of viable CD45+. D-F) Monocytes were defined as HLA-DR+CD11b+CD14+ cells of viable CD45+. G-I) Natural killer (NK) cells were defined as CD3-HLA-DR-CD8+NKG2A/C+NKp44- cells of viable CD45+. J-K) Macrophages were defined as HLA-DR+CD11b+CD14+CD68+ cells of viable CD45+. In all plots, each individual RM is shown with a different color and symbol. Timepoints are connected by lines. Baseline (BL) is an average of data collected at weeks -6, -4, -2, and 0 p.i. Inoculation with SIVmac239 at week 0 p.i. is indicated by a vertical purple dashed arrow. Inoculation with *P. fragile* at week 12 p.i. is indicated by a vertical blue dashed arrow. Antiretroviral therapy (ART) was initiated at week 8 p.i. and is indicated by the horizontal black dashed line. Antimalarial administration occurred between weeks 13 and 19 and is indicated by the horizontal green dashed line. α-Pf = anti-malarial treatment. Statistical significance at each timepoint compared to baseline was calculated using a mixed-effects analysis with the Geisser-Greenhouse correction and a Dunnett's multiple comparisons test, with individual variances computed for each comparison. Multiplicity-adjusted significant *P* values are shown above horizontal black bars.

Supplementary Figure 5

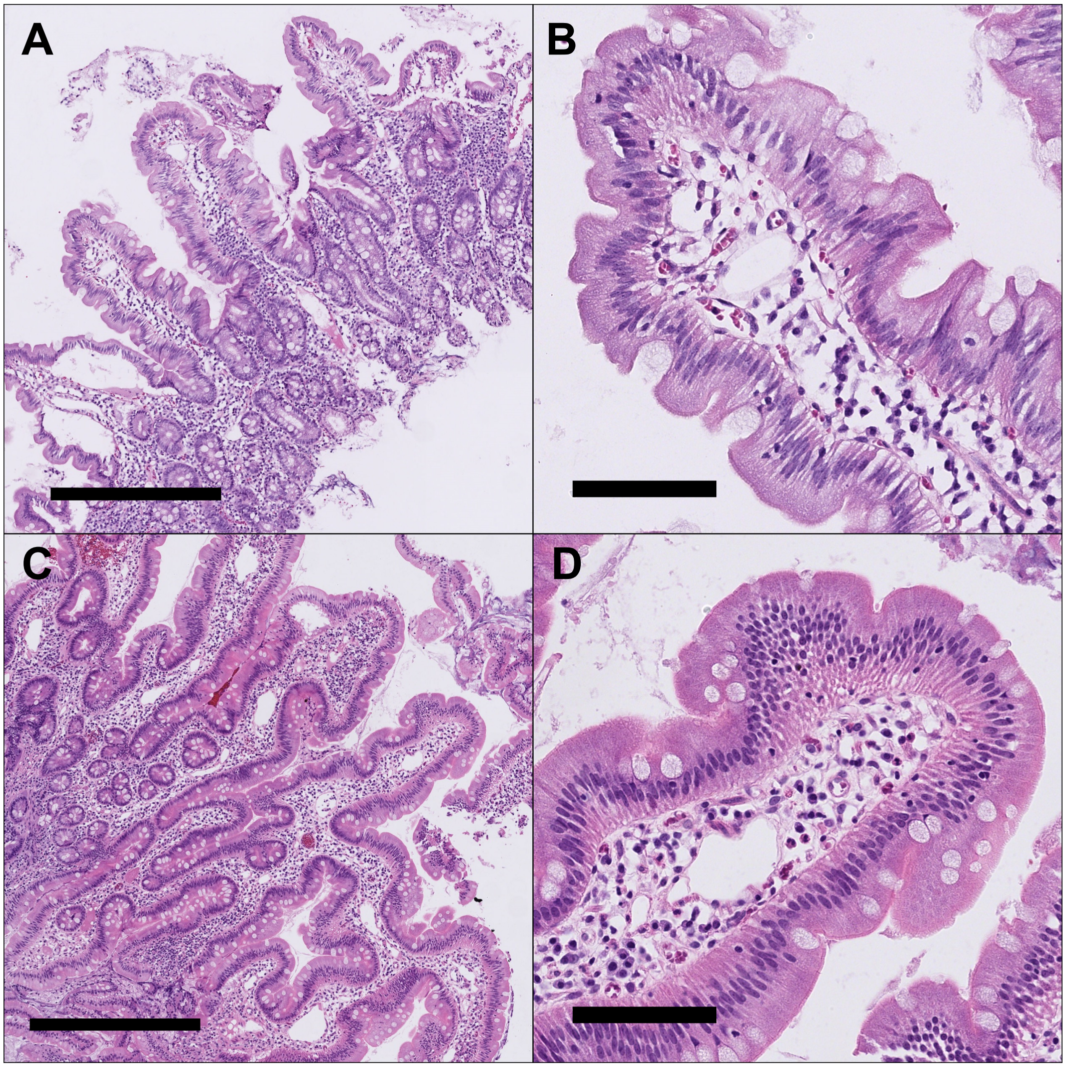

Duodenum histopathology. Duodenum histopathology was assessed via hematoxylin and eosin (H&E) staining. A-B) Representative images from RM21-0031. C-D) Representative images from RM23-0179. Mild inflammation was observed in the lamina propria of all animals. No significant differences were noted between animals or across time points. Bar= 500 µm (left) or 100 µm (right). The representative images for RM21-0031 and RM23-0179 were chosen from week 16 p.i. for their superior sample quality (orientation and depth).

PATHOGENS AND IMMUNITY
IN ASSOCIATION WITH CASE WESTERN RESERVE UNIVERSITY AND THE RICHARD J. FASENMYER FOUNDATION

Supplementary Table 1. Anti-malarial Treatments

**Timepoint**[1]		**Anti-malarial Treatment**					
Weeks post-SIV	Days post-*P. fragile*	**RM21 -0030**	**RM21 -0031**	**RM21 -0032**	**RM21 -0033**	**RM23 -0178**	**RM23 -0179**
**Week 13**	**9 d.p.i**	N/A	N/A	N/A	N/A	QS[2]	QS
**Week 13**	**10 d.p.i**	N/A	N/A	N/A	N/A	QS	QS
**Week 13**	**11 d.p.i**	N/A	N/A	N/A	N/A	QS	QS
**Week 13**	**12 d.p.i**	N/A	N/A	N/A	N/A	QS	QS
**Week 14**	**14 d.p.i**	QS*[3]	QS*	QS*	QS*	N/A	N/A
**Week 14**	**15 d.p.i**	Ch[4]	Ch	Ch	Ch	N/A	N/A
**Week 14**	**16 d.p.i**	Ch	Ch	Ch	Ch	N/A	N/A
**Week 14**	**17 d.p.i**	Ch	Ch	Ch	Ch	N/A	N/A
**Week 14**	**18 d.p.i**	Ch	Ch	Ch	Ch	N/A	N/A
**Week 15**	**22 d.p.i**	N/A	N/A	N/A	N/A	QS	QS
**Week 15**	**23 d.p.i**	N/A	N/A	N/A	N/A	QS	QS
**Week 15**	**24 d.p.i**	N/A	N/A	N/A	N/A	QS	N/A
**Week 15**	**25 d.p.i**	N/A	N/A	N/A	N/A	QS	N/A
**Week 15**	**26 d.p.i**	N/A	N/A	N/A	N/A	QS	N/A

Nemphos SM, Green HC, Prusak JE, Fell SL, Midkiff C, Rodgers A, Perret J, Goff K, Miller J, Varnado M, Didier K, Valencia N, Moström MJ, Tatum C, Barnes MB, Krzykwa CE, Rowe LA, Allers C, Grasperge B, De Paris K, Maness NJ, Kaur A, Londono-Renteria B, Blair RV, Manuzak JA. Gastrointestinal Mucosal Disruptions During ART-Treated SIV/*Plasmodium fragile* Co-Infection. *Pathogens and Immunity*. 2026;11(1):39–65. doi: 10.20411/pai.v11i1.854

**Week 17**	**37 d.p.i**	N/A	N/A	N/A	N/A	N/A	QS
**Week 18**	**42 d.p.i**	N/A	N/A	N/A	N/A	QS	N/A
**Week 18**	**43 d.p.i**	N/A	N/A	N/A	N/A	QS	N/A
**Week 18**	**44 d.p.i**	N/A	N/A	N/A	N/A	QS	N/A
**Week 18**	**45 d.p.i**	N/A	N/A	N/A	N/A	N/A	QS
**Week 18**	**46 d.p.i**	N/A	N/A	N/A	N/A	N/A	QS

[1]Anti-malarial treatment regimens by week and animal following *P. fragile* co-infection of ART-treated, SIV-infected RMs.

[2]QS indicates 150 mg of Quinine Sulfate.

[3] * indicates treatment given by oral gavage.

[4]Ch indicates 20 mg/kg of Chloroquine.

PATHOGENS AND IMMUNITY
IN ASSOCIATION WITH CASE WESTERN RESERVE UNIVERSITY AND THE RICHARD J. FASENMYER FOUNDATION

Supplementary Table 2. Myeloid Panel Flow Cytometry

Antibody[1]	Clone	Color	Company	Catalogue Number	Extracellular or Intracellular	Host
CD3	SP34	FITC	BD Biosciences	556611	Extracellular	Mouse IgG3, λ
CD20	2H7	FITC	BioLegend	302304	Extracellular	Mouse IgG2b, **κ**
CD66abce	TET2	PerCP-Vio700	Miltenyi	130-119-850	Extracellular	Mouse IgG2b, **κ**
CD49d	HP2/1	APC	Beckman Coulter	B01682	Extracellular	Mouse IgG1
CD206	19.2	AL700	Invitrogen	56-2069-42	Intracellular	Mouse IgG1, **κ**
CD11b	ICRF44	APC-Cy7	BD Biosciences	557754	Extracellular	Mouse IgG1, **κ**
Caspase 3	C92-605	V450	BD Biosciences	560627	Intracellular	Rabbit IgG
CD163	GHI/61	BV605	BD Biosciences	745091	Extracellular	Mouse IgG1, **κ**
CD16	3G8	BV650	BD Biosciences	563691	Extracellular	Mouse IgG1, **κ**
CD169	7-239	BV711	BD Biosciences	742995	Extracellular	Mouse IgG1, **κ**

Nemphos SM, Green HC, Prusak JE, Fell SL, Midkiff C, Rodgers A, Perret J, Goff K, Miller J, Varnado M, Didier K, Valencia N, Moström MJ, Tatum C, Barnes MB, Krzykwa CE, Rowe LA, Allers C, Grasperge B, De Paris K, Maness NJ, Kaur A, Londono-Renteria B, Blair RV, Manuzak JA. Gastrointestinal Mucosal Disruptions During ART-Treated SIV/*Plasmodium fragile* Co-Infection. *Pathogens and Immunity*. 2026;11(1):39–65. doi: 10.20411/pai.v11i1.854

HLA-DR	G46-6	BV785	BD Biosciences	564041	Extracellular	Mouse IgG2a, κ
CD68	Y1/82A	PE	BioLegend	333081	Intracellular	Mouse IgG2b, κ
CD86	2331	PE-CF594	BD Biosciences	562390	Extracellular	Mouse IgG1, κ
CD14	M5E2	PE-Cy5	BioLegend	301864	Extracellular	Mouse IgG2a, κ
CD11c	3.9	PE-Cy7	BioLegend	301608	Extracellular	Mouse IgG1, κ
CD45	D058-1283	BUV395	BD Biosciences	564099	Extracellular	Mouse IgG1, κ
CD62L	SK11	BUV496	BD Biosciences	750589	Extracellular	Mouse IgG2a, κ
CD123	7G3	BUV737	BD Biosciences	741769	Extracellular	Mouse IgG2a, κ

[1]Antibody name, clone, color, company, catalogue number, staining step, and host of extracellular antibodies used in flow cytometric staining.

Supplementary Table 3. Lymphoid Panel Flow Cytometry

**Antibody[1]**	**Clone**	**Color**	**Company**	**Catalogue Number**	**Extracellular or Intracellular**	**Host**
CD336	2.29	FITC	Milteny Biotec	130-118-542	Extracellular	Mouse IgG1
CD4	OKT4	PerCP-Cy5.5	Biolegend	317428	Extracellular	Mouse IgG2b, κ
a4b7	A4B7R1	APC	NHP Reagent Resources	N/A	Extracellular	Rhesus IgG1
Ki67	B56	AL700	BD Biosciences	561277	Intracellular	Mouse IgG1, κ
CD3	SP34-2	APC-Cy7	BD Biosciences	557757	Extracellular	Mouse IgG1, λ
Caspase 3	C92-605	V450	BD Biosciences	560627	Intracellular	Rabbit IgG
CD117	104D2	BV605	BioLegend	313218	Extracellular	Mouse IgG1, κ
CD16	3G8	BV650	BD Biosciences	563691	Extracellular	Mouse IgG1, κ
CD69	FN50	BV711	Biolegend	310944	Extracellular	Mouse IgG1, κ
HLA-DR	G46-6	BV786	BD Biosciences	564041	Extracellular	Mouse IgG2a, κ
ST2	Polyclonal	PE	R&D Systems	FAB5231P-100	Extracellular	Goat IgG

Nemphos SM, Green HC, Prusak JE, Fell SL, Midkiff C, Rodgers A, Perret J, Goff K, Miller J, Varnado M, Didier K, Valencia N, Moström MJ, Tatum C, Barnes MB, Krzykwa CE, Rowe LA, Allers C, Grasperge B, De Paris K, Maness NJ, Kaur A, Londono-Renteria B, Blair RV, Manuzak JA. Gastrointestinal Mucosal Disruptions During ART-Treated SIV/*Plasmodium fragile* Co-Infection. *Pathogens and Immunity*. 2026;11(1):39–65. doi: 10.20411/pai.v11i1.854

PATHOGENS AND IMMUNITY
IN ASSOCIATION WITH CASE WESTERN RESERVE UNIVERSITY AND THE RICHARD J. FASENMYER FOUNDATION

CD56	B159	PE-CF594	BD Biosciences	562289	Extracellular	Mouse IgG1, κ
CD127	R34.34	PE-Cy5	Beckman Coulter	A64617	Extracellular	Mouse IgG1
NKG2A/C	Z199	PE-Cy7	Beckman Coulter	B10246	Extracellular	Mouse IgG2b
CD45	D058-1283	BUV395	BD Biosciences	564099	Extracellular	Mouse IgG1, κ
CD8	SK1	BUV496	BD Biosciences	741199	Extracellular	Mouse IgG, λ
CCR5	3A9	BUV737	BD Biosciences	748873	Extracellular	Mouse IgG2a, κ

[1]Antibody name, clone, color, company, catalogue number, and host of extracellular antibodies used in flow cytometric staining.

PATHOGENS AND IMMUNITY
IN ASSOCIATION WITH CASE WESTERN RESERVE UNIVERSITY AND THE RICHARD J. FASENMYER FOUNDATION

Supplementary Table 4. Absolute Count Flow Cytometry

Antibody[1]	Clone	Color	Company	Catalogue Number	Host
CD3	SP34	FITC	BD Biosciences	556611	Mouse IgG3, λ
CD45	D058-1283	PerCP	BD Biosciences	558411	Mouse IgG1, κ
CD4	L200	APC	BD Biosciences	551980	Mouse IgG1, κ
CD8	SK1	V500	BD Biosciences	561618	Mouse IgG1, κ

[1]Antibody name, clone, color, company, catalogue number, and host of extracellular antibodies used in absolute count flow cytometric staining.

Pathogens and Immunity
In Association with Case Western Reserve University and The Richard J. Fasenmyer Foundation

Supplementary Table 5. MPO and CitH3 Staining Antibodies

Primary Antibody[1]	Host	Company	Catalogue Number	Dilution	Secondary Antibody	Color Development
MPO	Rabbit	Dako	A0398	1:1000	DISC. OmniMap Anti-Rb HRP RUO	Discovery Rhodamine
CitH3	Rabbit	Abcam	ab281584	1:400	DISC. OmniMap Anti-Rb HRP RUO	Discovery Cy5
DAPI	N/A	Invitrogen	D1306	1:10,000	N/A	N/A

[1]Primary antibody, host, company, catalogue number, dilution factor, secondary antibody, and color development used for identification of neutrophil extracellular trap (NET) forming granulocytes via fluorescent immunohistochemistry.

PATHOGENS AND IMMUNITY
IN ASSOCIATION WITH CASE WESTERN RESERVE UNIVERSITY AND THE RICHARD J. FASENMYER FOUNDATION

Supplementary Table 6. *P. fragile*/KK41 Dual Staining

**Primary Antibody[1]**	**Host**	**Company**	**Catalogue Number**	**Dilution**	**Secondary Antibody**	**Color Development**
P. fragile	N/A	ACD	1261269-C1	N/A	N/A	FITC
DapB	N/A	ACD	320759	N/A	N/A	FITC
KK41	Mouse	NIH	2317	1:50	Anti-mouse IgG1	Red Alexa 568
DAPI	N/A	Invitrogen	D1306	1:10,000	N/A	N/A

[1]Primary antibody, host, company, catalogue number, dilution factor, secondary antibody, and color development used for identification of the *P. fragile* 18s gene via in situ hybridization and SIVmac gp41 envelope protein (KK41) via fluorescent immunohistochemistry.
